# Supplementary figures and images for: vmTracking enables highly accurate multi-animal pose tracking in crowded environments
Source: PLoS Biol. 2025 Feb 10;23(2):e3003002. doi: 10.1371/journal.pbio.3003002 (PMC11845028; doi:10.1371/journal.pbio.3003002)

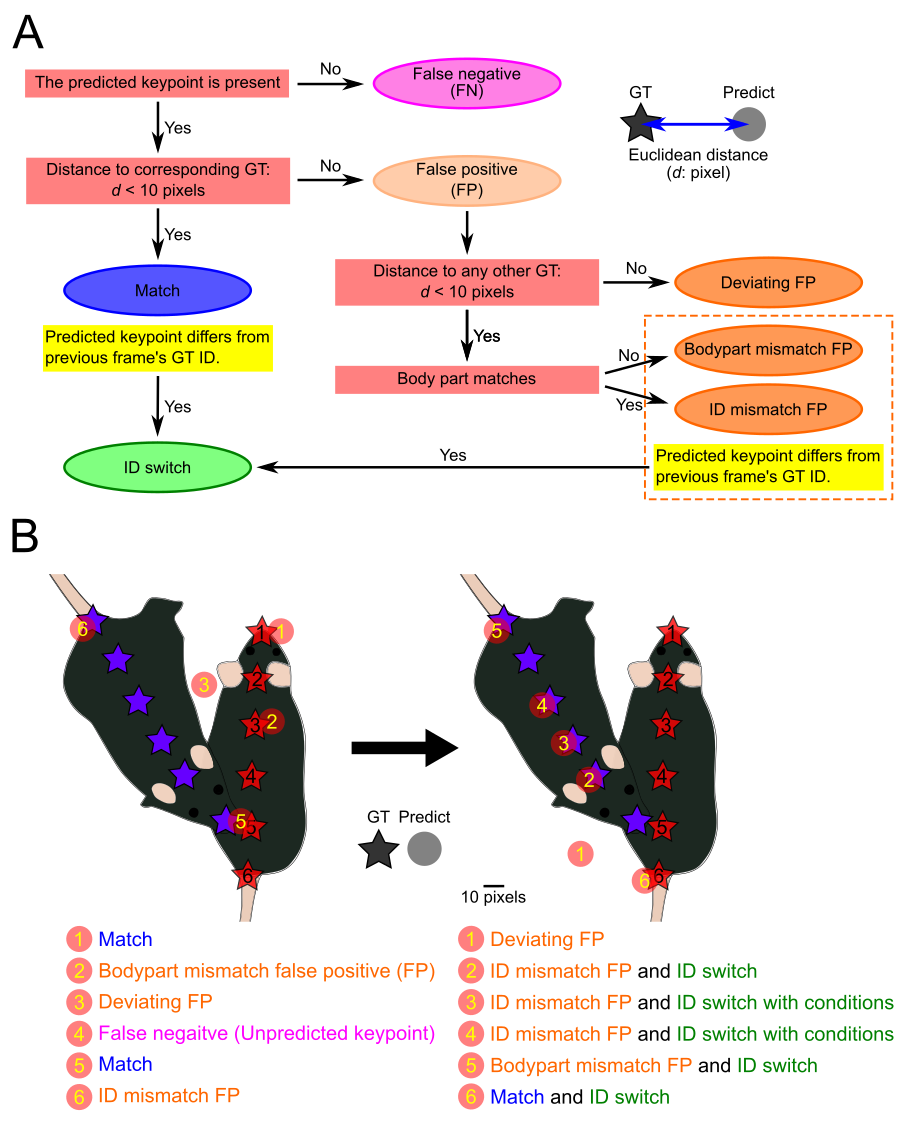

Supplement: S1 Fig — (A) Workflow of predicted keypoint classification. If a predicted point was within the threshold of 10 pixels from the corresponding ground truth (GT), it was considered a match; if a prediction was made but fell outside this threshold, it was counted as a false positive (FP); and if no predicted point was detected, it was classified as a false negative (FN). Furthermore, FPs were categorized by pattern as follows: FP when outside the threshold for all GT keypoints (Deviating FP); ID mismatch FPs when the body part was correct but assigned to an incorrect ID (ID mismatch FP); and body part mismatch FP when the predicted body part was incorrect, regardless of ID (Bodypart mismatch FP). When a predicted point was within the threshold of multiple GT keypoints, the GT keypoint classified as a match took priority; whereas when no match was identified, the nearest GT keypoint was used for evaluation. If any GT keypoint was within the threshold—such as in the case of a match, ID mismatch FP, or body part mismatch FP—but the ID differed from the previous frame (or an earlier frame if it was a FN and deviating FP), this was classified as an ID switch. Red stars (ground truth) and red circles (predictions) correspond to each other. (B) Example of predictive keypoint classification. For the left of the arrow, 1: accurately predicts a location within 10 pixels of the corresponding GT (Match); 2: predicts a location more than 10 pixels away from the corresponding GT and within 10 pixels of a non-corresponding GT (Bodypart mismatch FP); 3: does not predict within 10 pixels of all GT (Deviating FP); 4: shows no predicted keypoints (FN); 5: is within 10 pixels of both the corresponding and non-corresponding GTs, with the corresponding GT being prioritized (Match); and 6: predicts a location more than 10 pixels away from the corresponding GT and within 10 pixels of a GT with a different ID but matching body part (ID mismatch FP). However, as ID switches are based on changes from the [file pbio.3003002.s001.tiff]

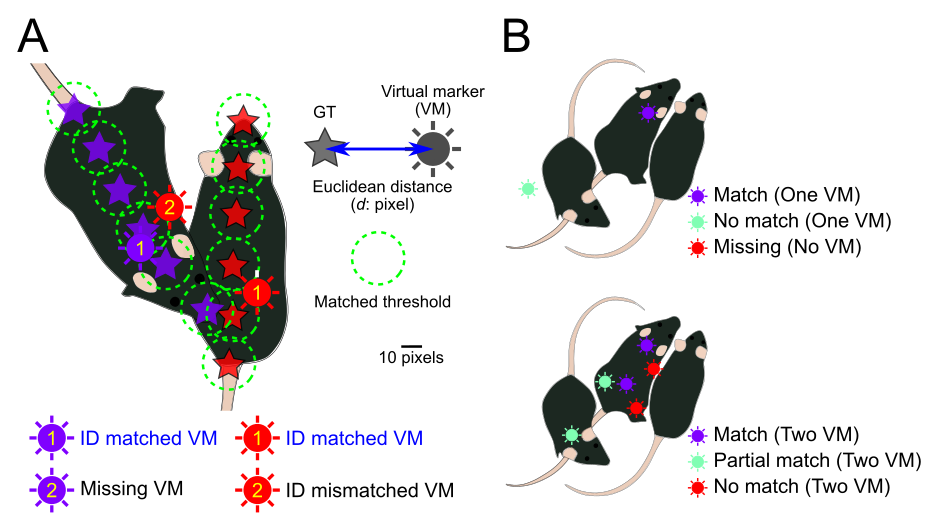

Supplement: S2 Fig — (A) Concept of ID matched virtual markers, which indicates whether a virtual marker’s ID match was based on the distance (d: pixels) between the virtual marker points indicated by radial circles and the ground truth indicated by stars. If any ground truth keypoints with the same ID as the virtual marker were within 10 pixels of the corresponding virtual marker, the virtual marker was considered to have an ID match (as shown in the figure for the red and purple virtual markers labeled “1”). Conversely, if no ground truth keypoints with the same ID as the virtual marker were within 10 pixels, the virtual marker was considered to have an ID mismatch (as shown in the figure for the absent purple virtual marker labeled “2”). Additionally, if virtual markers were absent due to missing predictions in markerless multi-animal pose tracking, these were treated as instances of ID mismatch, as they represent missing virtual markers that should have been present (as shown in the figure for the purple virtual marker labeled “2”). The proportion of ID-match virtual marker points for each scene was calculated and used for the correlation analysis with the accuracy of vmTracking. (B) Classification of virtual marker assignment patterns in the mouse tracking experiment, 2 keypoints in markerless multi-animal tracking were designated as virtual markers. For frames where both keypoints are present, virtual marker assignments are categorized as follows: both IDs match (Match (Two VM)), only 1 ID matches (Partial match (Two VM)), and neither ID matches (No match (Two VM)). When only 1 keypoint is present (the other being missing), it is categorized based on whether its ID matches (Match (One VM)) or does not match (No match (One VM)), and when both keypoints are missing, it is classified as Missing (No VM). This classification was performed for each mouse in each frame, and the frequency of each category was calculated for each scene. (TIFF) [file pbio.3003002.s002.tiff]

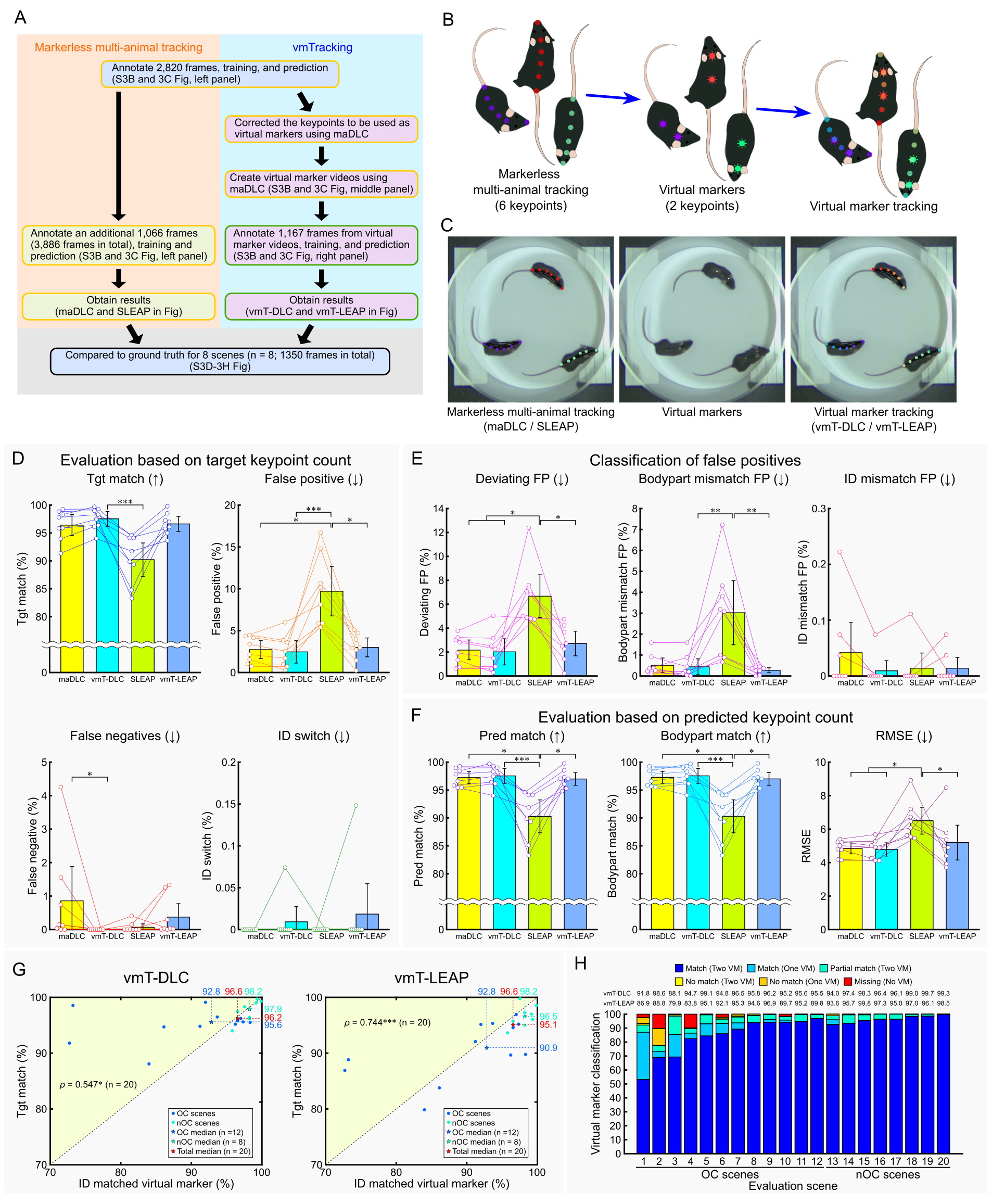

Supplement: S3 Fig — (A) Overview of the verification process. The yellow box in the schematic represents the processes using multi-animal tracking tools, while the green box represents the processes using single-animal tracking tools. (B) Schematic of virtual marker creation. Six keypoints per mouse were tracked, with 2 designated as virtual markers. (C) Examples of markerless multi-animal pose tracking (left), virtual marker (middle), and virtual marker tracking (right) in a non-occlusion-crowded (nOC) scene. (D–F) Various percentage-based metrics were compared across multi-animal DeepLabCut (maDLC), virtual marker tracking with DeepLabCut (vmT-DLC), Social LEAP (SLEAP), and virtual marker tracking with SLEAP (vmT-LEAP), based on manually generated ground truth (GT) data. (D) The evaluation was conducted as a percentage of the target keypoints, with the total number of GT keypoints as the denominator. The percentages of matches (Tgt match), false negatives, false positives, and ID switches were calculated based on the number of GT keypoints. (E) False positives from (D) were classified into deviations exceeding the threshold from all GT (Deviating FP), mismatches in predicted body parts (Bodypart mismatch FP), and cases where the predicted body part was correct but the ID was incorrect (ID mismatch FP). (F) Evaluation was conducted as a percentage of the predicted keypoints, excluding FNs, using the total predicted keypoints as the denominator, and the metrics included matches (Pred match), body part matches (Bodypart match), irrespective of ID, and root mean square error (RMSE). Arrows indicate whether higher or lower values are better. Data are shown as the mean ± 95% confidence interval (n = 8 scenes), with individual measurements shown as plots. Statistical analysis was performed using the Friedman test with Bonferroni correction. (G) Scatter plots illustrating the relationship between ID matched virtual markers and Tgt match for vmT-DLC (left) and vmT-LEAP (right). The plots are [file pbio.3003002.s003.tiff]

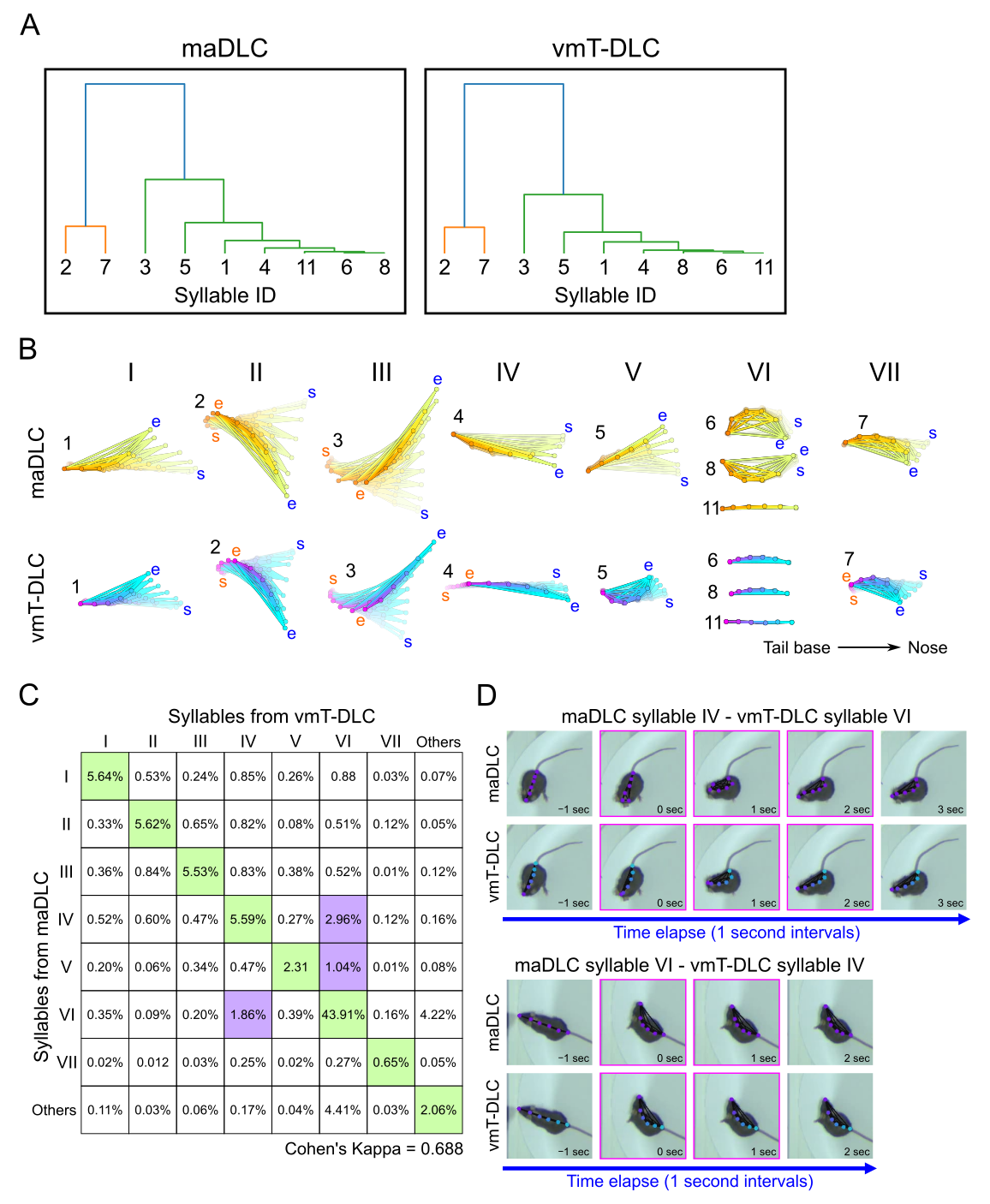

Supplement: S4 Fig — (A) Similarity dendrogram of syllables obtained from keypoint-MoSeq analysis on the tracking results of multi-animal DeepLabCut (maDLC) and virtual marker tracking with single-animal DeepLabCut (vmT-DLC) across all 112,991 frames. The syllable IDs correspond to the Arabic numerals in (B). (B) Behavioral syllables obtained from maDLC and vmT-DLC. Here, syllables 6, 8, and 11 were grouped and processed as nearly immobile patterns based on the similarity dendrogram results and syllable patterns. Each syllable represents a 1-s behavioral pattern, with the left side of each plot representing the base of the tail and the right side representing the head (snout). In the figure, “s” denotes the start point of the behavior, and “e” denotes the end point. Blue text indicates head-side movement, while orange indicates tail-base movement. When there was minimal change at the start or end points, these letters were omitted. (C) Confusion matrix for syllable similarity obtained from maDLC and vmT-DLC. The Roman numerals correspond to the 7 syllables in (B), and minor syllables not included in I–VII are grouped as “Others.” The matrix shows the frequency of syllable number combinations assigned to each frame by maDLC and vmT-DLC (for example, frames where syllable I was assigned by both maDLC and vmT-DLC make up 5.64% of the total). Cells shaded in light green indicate frames where the same syllable was assigned by both maDLC and vmT-DLC, while cells shaded in light purple indicate cases where different syllables were assigned by maDLC and vmT-DLC, with a frequency of 1% or more (excluding cases that involve “Others”). The value below the matrix represents Cohen’s kappa coefficient as an indicator of agreement. (D) Examples of tracking by maDLC and vmT-DLC in scenes where the assigned syllables did not match and this discrepancy persisted for more than 2 s (60 frames). The upper row shows a scene where maDLC consistently assigned syllable VI while vmT-DLC assigned syllable IV. The [file pbio.3003002.s004.tiff]

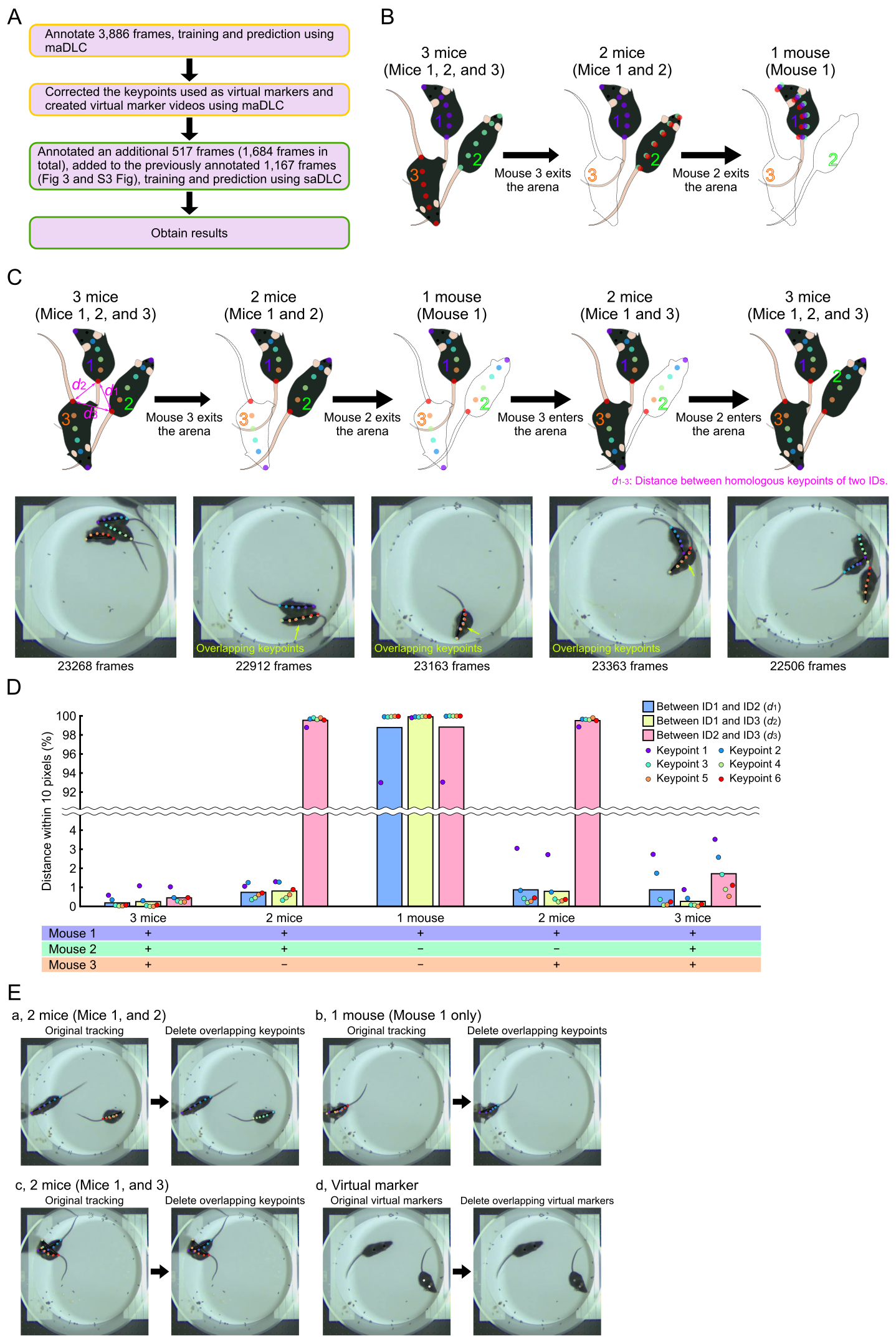

Supplement: S5 Fig — (A) Overview of the verification process. The yellow box in the schematic represents the processes using multi-animal tracking tools, while the green box represents the processes using single-animal tracking tools. (B) Schematic diagram of the annotation method. The outlined mouse in the schematic indicates that the mouse was removed from the arena and is absent in the frame. In the case of 3 mice (left), 6 standard annotations were made for each mouse. In the case of 2 mice (center), keypoints for Mouse 2 and Mouse 3 were annotated on each body part of the same mouse. In the case of one mouse (right), keypoints for all mice were annotated on the body parts of that single mouse. Here, the labels are color-coded by individual. (C) Schematic diagram (top) of an experiment that altered the number of mice in the video frame by introducing or removing mice from the arena, and examples of tracking photos during this procedure (bottom). To verify tracking of other mice with leftover keypoints due to the absence of some mice, distances (d: pixels) between identical keypoints for 3 IDs were calculated for each keypoint. The stray keypoints indicated above the outlined mouse, which represents absence in the frame in the top schematic, were evaluated to determine whether they could predict another mouse in the arena, following the annotation method described in (B). Here, the labels are color-coded by body part. (D) If the distance between identical keypoints was within 10 pixels, the 2 ID keypoints were considered overlapping, and the frequency of such frames was calculated for each keypoint and ID pair combination. The diagram illustrates changes across different experimental conditions, with plots indicating the frequency for each keypoint and bars representing each ID pair. The “+” below the diagram indicates the presence of mice in the arena, while “−” indicates their absence. Statistical analysis was conducted using the Friedman test for comparisons within each condition [file pbio.3003002.s005.tiff]

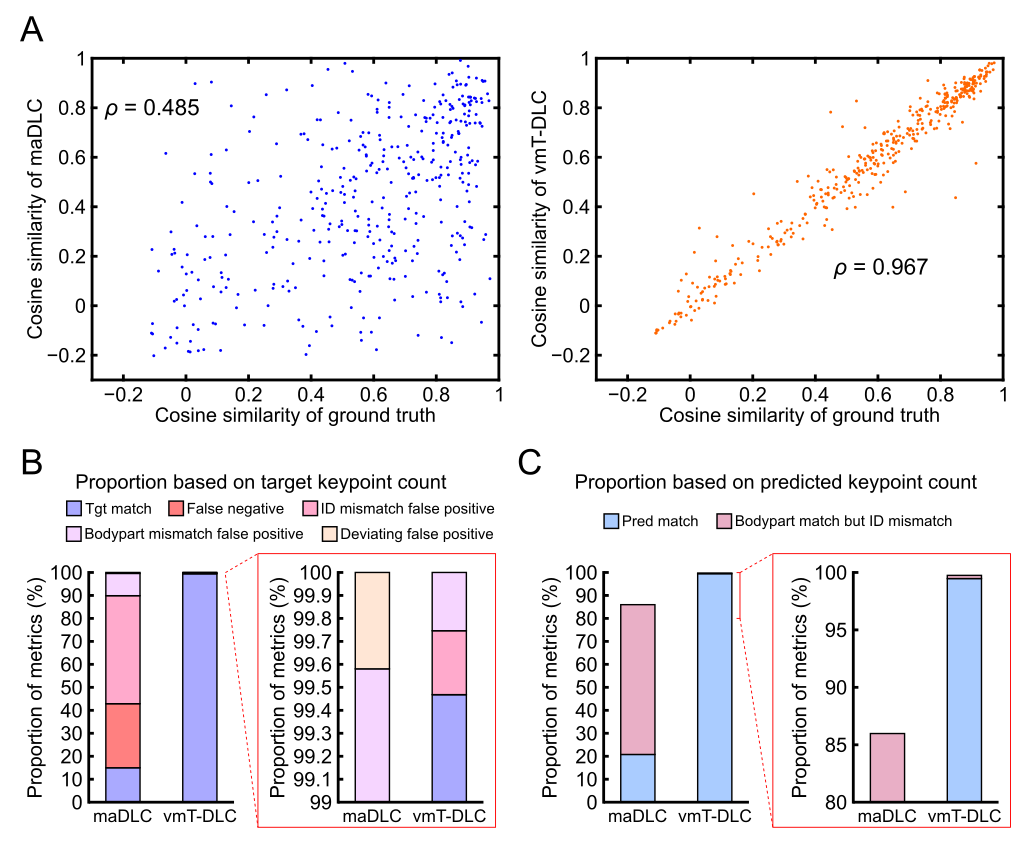

Supplement: S6 Fig — (A) Correlation of cosine similarity with the ground truth (GT). The GT consisted of 410 frames. The scatter plot on the left shows the cosine similarity between the ground truth and maDLC (ρ = 0.485, n = 407), while the plot on the right shows the cosine similarity between the ground truth and vmT-DLC (ρ = 0.967, n = 407). Correlation analysis was conducted using Spearman’s rank correlation. (B) Stacked bar graph showing ground truth-based matches (Tgt match), false negatives, ID mismatch false positives (FPs), body part mismatch FPs, and deviating FPs for multi-animal DeepLabCut (maDLC) and virtual marker tracking with single-animal DeepLabCut (vmT-DLC). The graph within the red box is an enlarged view of the section with metrics showing low percentages. (C) Stacked bar graph of predicted keypoint matches (Pred match) and cases where body parts matched but the ID was mismatched (Bodypart match but ID mismatch). These values are derived from the data associated with the video evaluated in Fig 7. The data underlying these analyses are provided in S1 Data. Additionally, these figures can be reproduced using the MATLAB data and code available in the Zenodo repository (https://doi.org/10.5281/zenodo.14545410). (TIFF) [file pbio.3003002.s006.tiff]

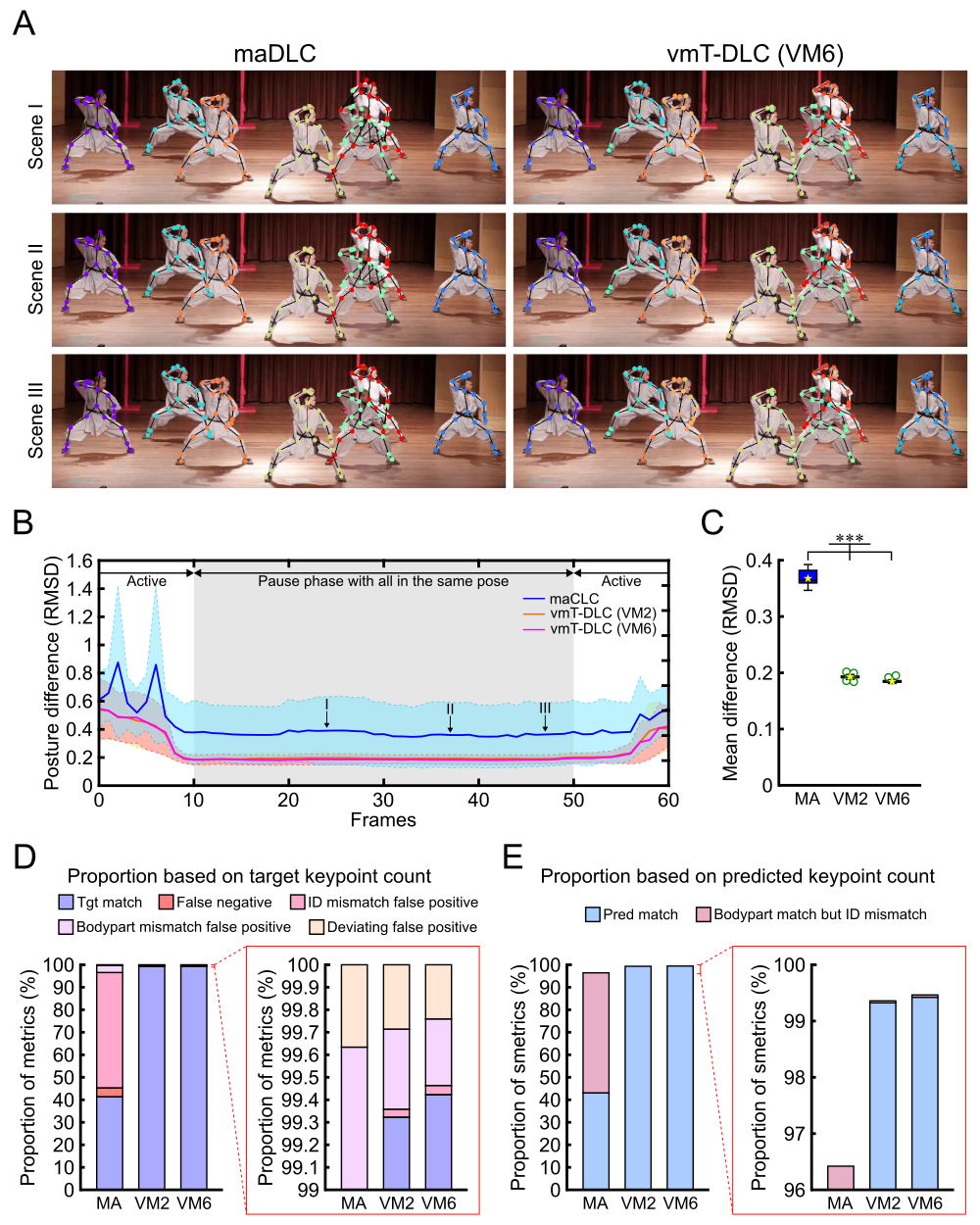

Supplement: S7 Fig — (A) Tracking scenes using multi-animal DeepLabCut (maDLC) for markerless tracking and single-animal DeepLabCut for virtual marker tracking (vmT-DLC), with 3 examples where 7 dancers are posed similarly and remain stationary for 2 s. The vmT-DLC results are based on the six-point virtual marker video (VM6). (B) Time series variation of RMSD over 3 s, including approximately 2 s of pause scenes. The shaded area along the plot line indicates standard deviation. The gray shaded area represents the pause scenes. I to III correspond to scenes I to III in (A). (C) Box plots of RMSD values for maDLC with markerless video (MA) and vmT-DLC with two-point virtual marker video (VM2) and VM6, calculated for all frames over a 2-s period, are presented. The bottom of the box represents the first quartile (Q1), the top represents the third quartile (Q3), the line inside the box indicates the median, the ends of the whiskers represent the maximum and minimum values excluding outliers, green circular markers denote outliers (values beyond 1.5 times the interquartile range from Q1 or Q3), and yellow star markers represent the mean. Statistical analysis was performed using the Friedman test with Bonferroni correction (n = 41). *: p < 0.05, **: p < 0.01, ***: p < 0.001. (D) Stacked bar graph showing ground truth (GT)-based matches (Tgt match), false negatives, ID mismatch false positives (FPs), body part mismatch FPs, and deviating FPs for MA, VM2, and VM6. The graph within the red box is an enlarged view of the section with metrics showing low percentages. (E) Stacked bar graph of predicted keypoint matches (Pred match) and cases where body parts matched but the ID was mismatched (Bodypart match but ID mismatch). The GT consisted of 150 frames. The data underlying these analyses are provided in S1 Data. Additionally, these figures can be reproduced using the MATLAB data and code available in the Zenodo repository (https://doi.org/10.5281/zenodo.14545410). (TIFF) [file pbio.3003002.s007.tiff]

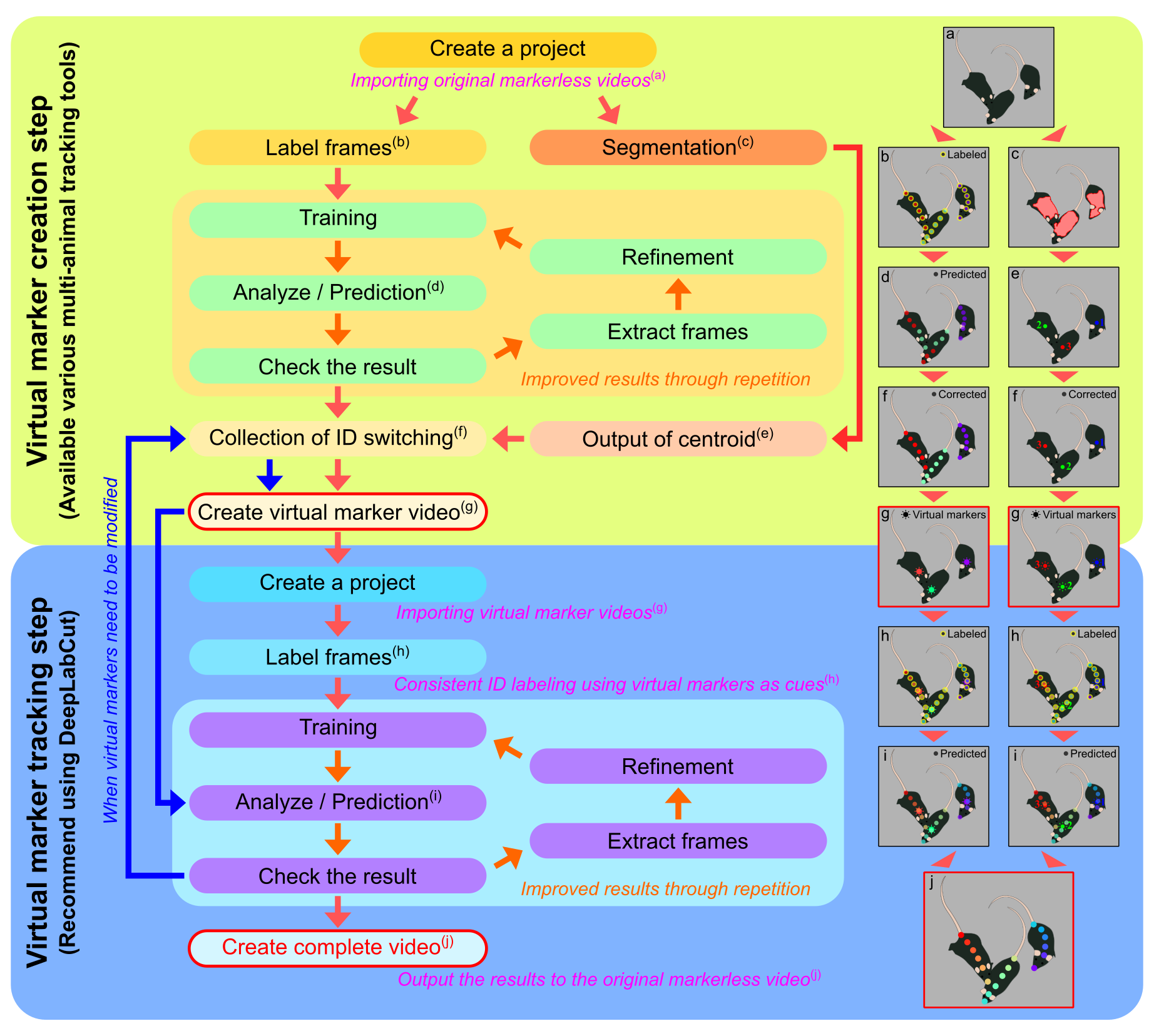

Supplement: S8 Fig — The vmTracking process consists of 2 major steps: creating the virtual marker video and tracking the virtual marker video. In the virtual marker creation step, a multi-animal tracking tool, such as multi-animal DeepLabCut (maDLC), Social LEAP (SLEAP), or idtracker.ai, is applied to the markerless video to track the animals. The tracking results are then corrected to ensure consistent ID assignment throughout the video, and the corrected tracking points are output as a new video. This video is called the virtual marker video. Thus, virtual markers are identification markers derived from the results of multi-animal tracking and do not physically exist. In the virtual marker tracking step, annotations are made so that consistent IDs are assigned to each individual across frames using the virtual markers as identification cues. Tracking is then performed using single-animal DeepLabCut, not maDLC. The resulting labels are applied to the original markerless video, producing a final tracking video without the virtual markers. (TIFF) [file pbio.3003002.s008.tiff]

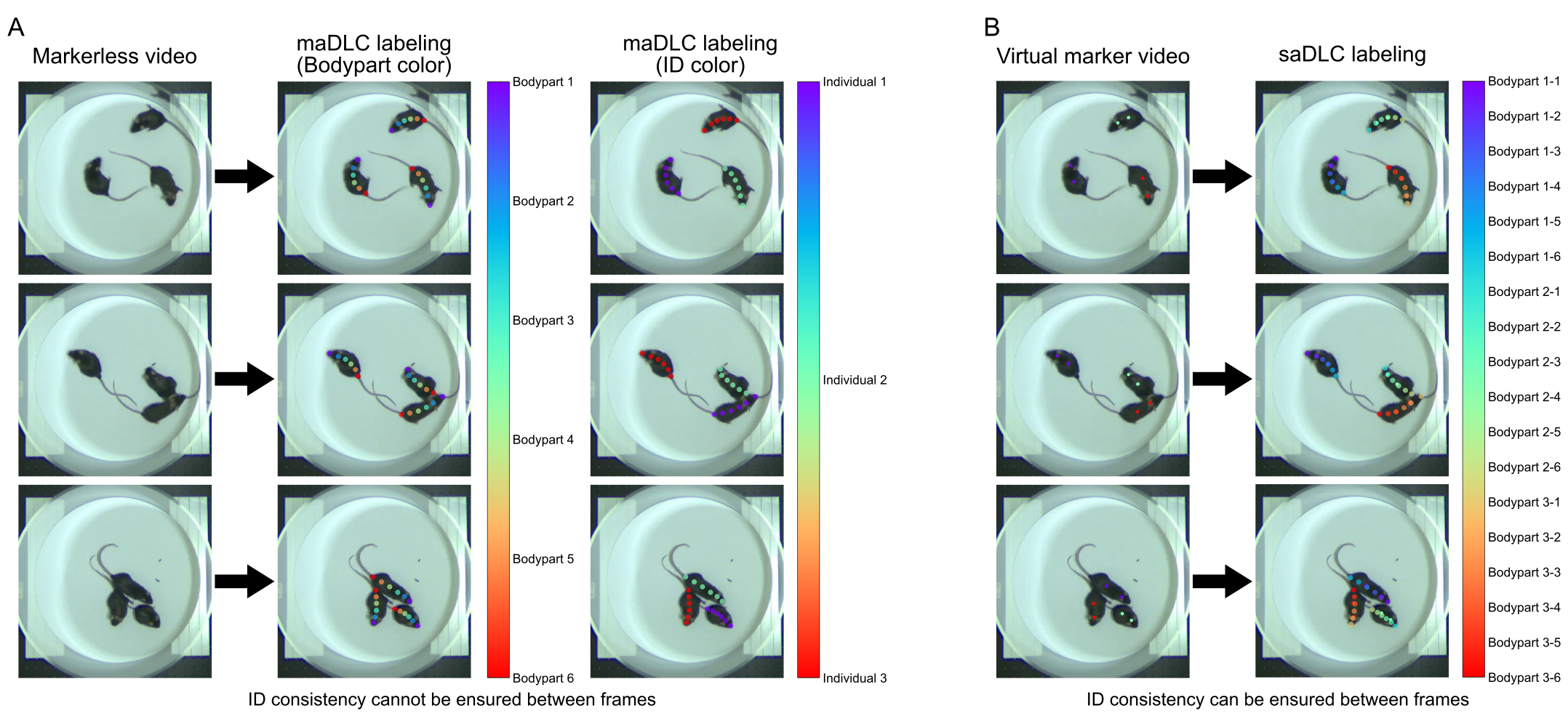

Supplement: S9 Fig — (A) Example of annotations by multi-animal DeepLabCut (maDLC) for markerless videos. In maDLC, it is possible to display using either body part color or ID color modes, but in DeepLabCut 2.2, ID color does not appear to be displayed during the initial label frame step. In DeepLabCut 2.3’s napari-deeplabcut, the display mode can be switched using the “F” key. While it is essential to annotate all body parts for every individual, it does not matter which ID is assigned to which individual if the relationship between the individual and the ID cannot be determined in each frame. (B) Example of annotations by single-animal DeepLabCut (saDLC) for virtual marker videos. Since saDLC is designed for a single individual, the annotations assume that all body parts belong to one individual. The annotations are made so that consistent IDs are assigned to the same individual across frames, using the virtual markers as identification cues. (TIFF) [file pbio.3003002.s009.tiff]

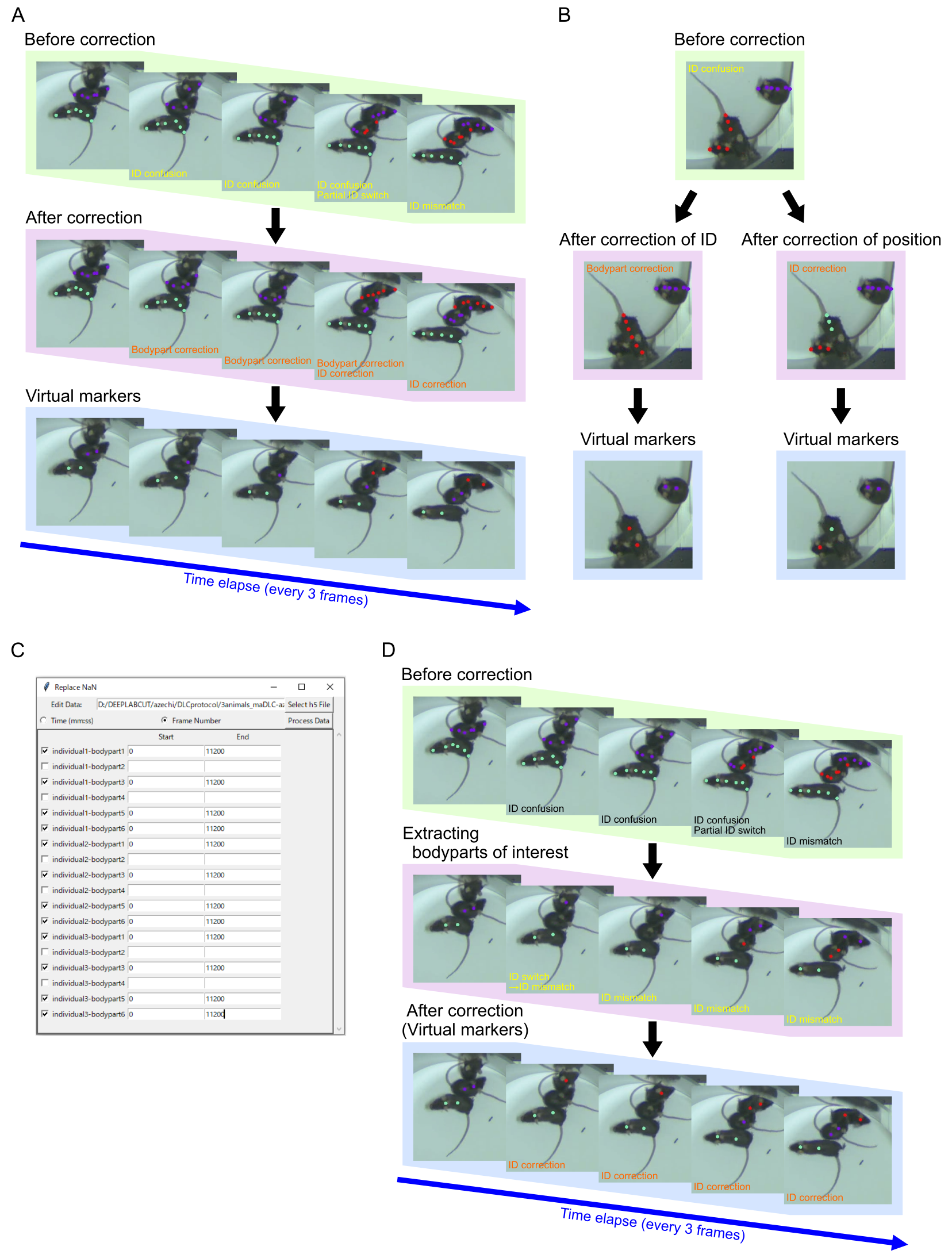

Supplement: S10 Fig — (A) When ID switching (including partial switching) occurs, it is corrected. In this example, body parts that switched IDs (where purple labels were assigned across multiple individuals) were first adjusted so that all body parts were associated with a single individual. Then, the overall ID was corrected to ensure consistency throughout the video. These corrections can be made in DeepLabCut (DLC) using the “Refine tracklets” function (specific instructions available at: https://www.youtube.com/watch?v=bEuBKB7eqmk) [35]. (B) When a single ID label spans multiple individuals, 2 correction methods can be applied: adjusting body parts so that the label with the same color is assigned to one individual (left), or adjusting the ID to assign unique colors (IDs) to each individual (right). The choice of correction method affects the resulting virtual markers. While it is difficult to determine which approach is universally best, choosing a method that avoids biased ID omissions is recommended. (C) Screenshot of a Python-based GUI created to delete specified keypoints and data ranges from coordinate data files (in h5 format) obtained from DLC, replacing them with NaN (no data). Keypoints and data ranges for deletion can be specified by a combination of fps and time or by frame number. This tool enables users to retain only the keypoints designated as virtual markers and make necessary adjustments for virtual marker creation. (D) Corrections using the above-mentioned Python code when retaining only keypoints 2 and 4 in the same data as (A). In this example, the task is completed by correcting only ID switches, as non-virtual marker keypoints were removed. Clarifying which keypoints require correction is expected to improve the efficiency of the correction process. (TIFF) [file pbio.3003002.s010.tiff]

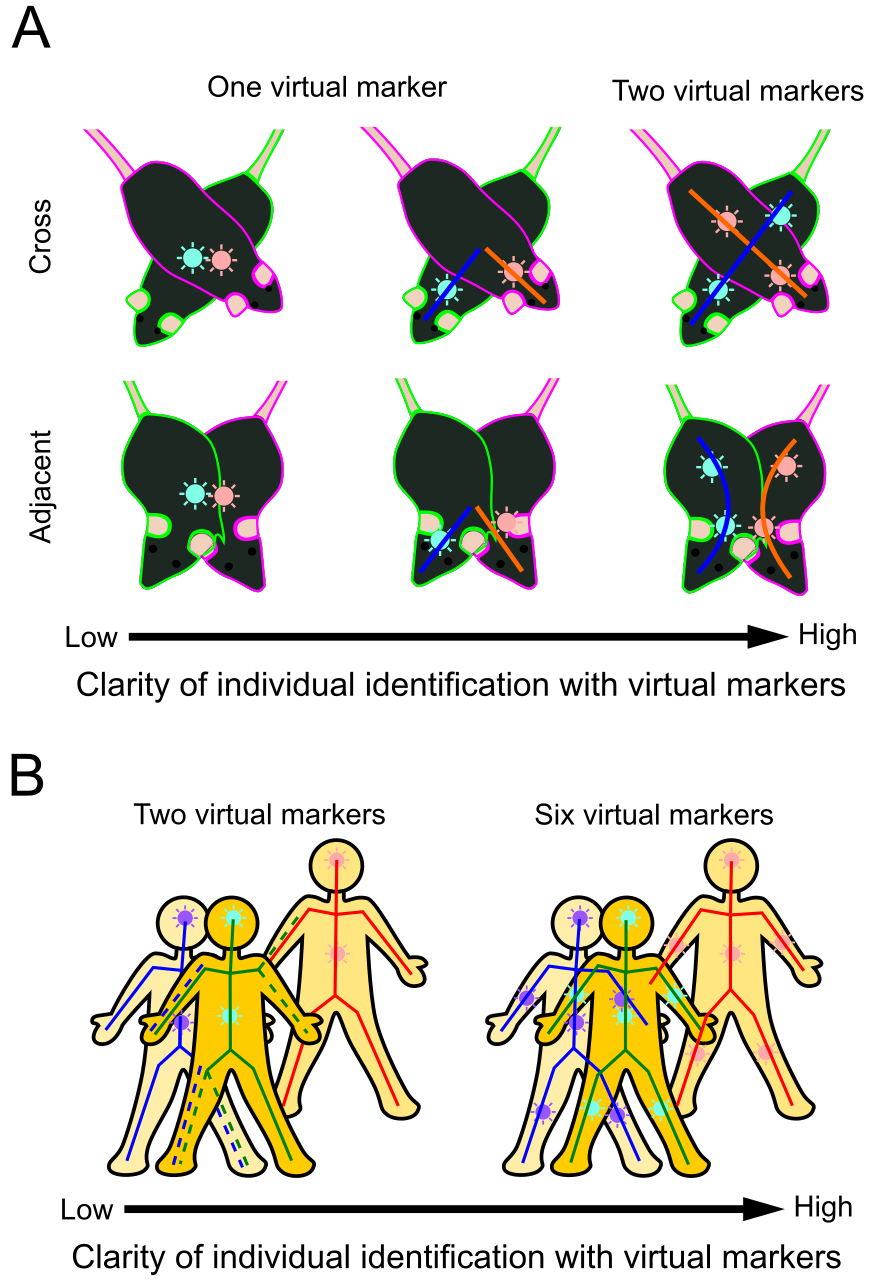

Supplement: S11 Fig — (A) Comparison of cases where there is 1 virtual marker versus 2 virtual markers per mouse. The blue and orange solid lines represent the body axis that can be identified using the virtual markers. When one point is placed at the center of each mouse’s body, it becomes difficult to clearly distinguish between mice that are overlapping or adjacent (left). When one point is placed near the head, it becomes possible to identify individuals by their head position, but the area from the body to the tail cannot be clearly distinguished (middle). When 2 points are placed, one near the head and one near the tail, the 2 mice can be clearly distinguished (right). (B) Comparison of cases where there are 2 virtual markers versus 6 virtual markers per human. The blue, green, and red solid lines represent the predicted skeletons based on the virtual markers. When 2 virtual markers are placed on the head and abdomen of each individual, there is a high possibility that the arms and legs will be confused with those of other individuals (as indicated by the dotted lines). On the other hand, placing 6 virtual markers, including on the arms and legs, is expected to make it easier to detect the entire body, including the limbs, with the correct ID. (TIFF) [file pbio.3003002.s011.tiff]

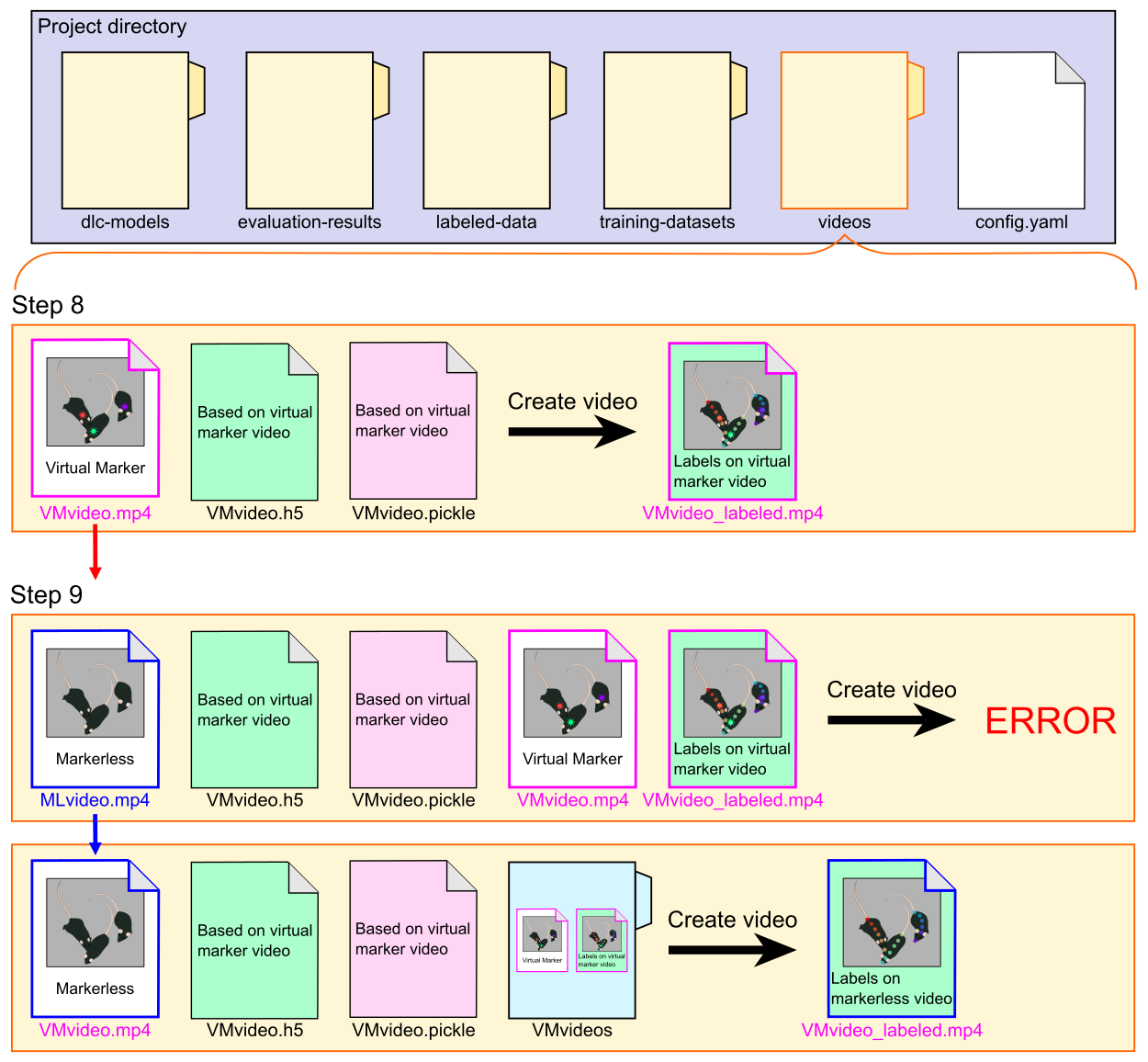

Supplement: S12 Fig — The top section of the figure is a schematic of the file structure within the project directory, followed by a schematic of the “videos” directory. When the results obtained from vmTracking are output to a video using the standard procedure, a video is created where the predicted labels are overlaid on the virtual marker video (Step 8). To apply the vmTracking results to a video without virtual markers, the results must be output to the markerless original video, and for this, the “videos” directory within the project directory needs to be properly prepared. If you simply place the markerless original video in the “videos” directory and attempt to create a video, an error will occur, and the video will not be generated (upper part of Step 9). If the videos used in Step 8 (the virtual marker video and the video with the overlaid results) are still in the “videos” directory, you should either rename these files or move them to a different directory (lower part of Step 9). Additionally, you need to rename the markerless original video appropriately. Since DeepLabCut processes files based on filename dependencies, the filename must correspond to the h5 and pickle files. Usually, changing the same filename as the virtual marker video used in vmTracking should work (lower part of Step 9). The Step numbers in the figure correspond to the steps in S1 Protocol. (TIFF) [file pbio.3003002.s012.tiff]
